# Inflammation dependent mTORC1 signaling interferes with the switch from keratinocyte proliferation to differentiation

**DOI:** 10.1371/journal.pone.0180853

**Published:** 2017-07-10

**Authors:** Claudia Buerger, Nitesh Shirsath, Victoria Lang, Alina Berard, Sandra Diehl, Roland Kaufmann, Wolf-Henning Boehncke, Peter Wolf

**Affiliations:** 1 Department of Dermatology, Venerology and Allergology, Clinic of the Goethe University, Frankfurt am Main, Germany; 2 Department of Dermatology, Medical University of Graz, Graz, Austria; 3 Department of Dermatology and Venereology, Geneva University Hospital, Geneva, Switzerland; 4 Department of Pathology and Immunology, Faculty of Medicine, University of Geneva, Geneva, Switzerland; NYU Langone Medical Center, UNITED STATES

## Abstract

Psoriasis is a frequent and often severe inflammatory skin disease, characterized by altered epidermal homeostasis. Since we found previously that Akt/mTOR signaling is hyperactivated in psoriatic skin, we aimed at elucidating the role of aberrant mTORC1 signaling in this disease. We found that under healthy conditions mTOR signaling was shut off when keratinocytes switch from proliferation to terminal differentiation. Inflammatory cytokines (IL-1β, IL-17A, TNF-α) induced aberrant mTOR activity which led to enhanced proliferation and reduced expression of differentiation markers. Conversely, regular differentiation could be restored if mTORC1 signaling was blocked. In mice, activation of mTOR through the agonist MHY1485 also led to aberrant epidermal organization and involucrin distribution. In summary, these results not only identify mTORC1 as an important signal integrator pivotal for the cells fate to either proliferate or differentiate, but emphasize the role of inflammation-dependent mTOR activation as a psoriatic pathomechanism.

## Introduction

To maintain homeostasis of the healthy epidermis keratinocyte stem cells divide asymmetrically, leave the basal layer and successively develop into the spinous, granular and corneal layers, characterized by ordered expression of keratins and other marker such as involucrin, loricrin, filaggrin or transglutaminase [1]. Upon maturation, keratinocytes undergo a form of programmed cell death and are shed as corneocytes [2]. The balance between keratinocyte proliferation and differentiation is tightly regulated, but is deregulated in certain skin diseases such as psoriasis. Psoriasis is a chronic inflammatory skin disease presenting with red scaly plaques, mostly on the head, trunk and extensor sites of arms and legs [3]. These lesions are characterized by thickened, irregular stratum corneum with parakeratosis, epidermal thickening with acanthosis and absence of the granular layer. This is caused by hyperproliferating keratinocytes that are unable to properly initiate the epidermal differentiation program [4].

The molecular mediators and intracellular signaling pathways of the inflammatory psoriatic process involving Th17/Th22 cells and their effector cytokines acting on keratinocytes are well understood [4]. However, despite increasing identification of deregulated signal mediators such as STAT1 and 3, kinases of the MAPK family, PKC isoforms as well NF-kB [5–9], a comprehensive concept of the signaling pathways governing epidermal homeostasis and its alterations in diseases such as psoriasis has yet to be established.

Previously we found that inflammation dependent dysregulation of the PI3-K/Akt cascade interferes with the equilibrium between keratinocyte proliferation and differentiation and potentially contributes to the pathogenesis of psoriasis [10]. An important effector of PI3-K/Akt via TSC1/2 and the small GTPase Rheb [11] is the mTOR signaling pathway. The mTOR kinase, existing in two different multi-protein complexes (mTORC1 and 2), plays a central role in regulating cell growth and proliferation and is frequently dysregulated in different tumors [12], notably as well in epidermal tumors [13].

Active mTORC1- consisting besides the mTOR kinase itself, of Raptor and PRAS40 as well as other regulatory proteins [12]—phosphorylates proteins such as 4E-BP1 and S6 kinase 1 (S6K1), which in turn phosphorylates the ribosomal protein S6. 4E-BP1 and S6 are involved in protein biosynthesis through the regulation of translation [14]. In addition mTORC1 is able to promote through the activation of transcription factors lipid biogenesis, energy metabolism and repress autophagy [15]. The function of mTORC2 is less defined. It phosphorylates Akt at S473 and other AGC-kinases, contributes to cytoskeleton reorganization and is potentially involved in the regulation of cell tugor [16].

Our group previously reported for the first time an increase in mTOR expression and phosphorylation in psoriatic skin as well as hyperactivation of S6K1 and the ribosomal protein S6 [17]. It is currently hypothesized that the PI3-K/Akt/mTOR cascade plays a role in the pathogenesis of psoriasis by regulating the function of immune cells as well as intrinsic alterations within the epidermis (reviewed in [18]). Thus, we aimed at deciphering the contribution of mTORC1 signaling to epidermal homeostasis and its pathogenic role in the psoriatic epidermis.

We could show that in healthy keratinocytes Akt/mTOR signaling was deactivated, when differentiation was progressing. In contrast, under inflammatory conditions such as psoriasis, cytokines induced aberrant activation of the mTOR cascade. This contributed to the induction and/or maintenance of the psoriatic phenotype through the initiation of proliferation and blockade of proper differentiation, thus pointing towards mTOR as a potential target for therapeutic intervention in psoriasis.

## Materials and methods

### Chemicals and antibodies

All chemicals were purchased from Sigma unless stated otherwise. Rapamycin, Wortmannin, LY294002 and MHY1485 were from Calbiochem. Torin was purchased from Tocris Bioscience. Cytokines were obtained from Peprotech.

Phospho-specific (P-mTOR S2448 #5536, P-mTOR S2448 #2976, P-PRAS T246 #2997, P-S6 S235/6 #2211, P-Akt S473 #4060, P-ERk1 T202/Y204 #4370, P-p38MAPK T180/Y182 #4511, and corresponding pan antibodies (PRAS #2691, Akt #2938, S6 #2217) Rheb antibody (AP53656PU-N) was from Acris and tubulin #2128 antibody was from Cell Signaling Technology. Raptor antibody (sc27744) was from Santa Cruz, actin antibody (A1978) was from Sigma, involucrin antibody (ab20202) and β1-integrin antibody (ab30394) were from Abcam and filaggrin (PRB-417P-100) was from Convance. Ki-67 (MB67) antibody was obtained from Novus Biologicals and Keratin6 antibody (Ks6.KA12) from Thermo Scientific. Involucrin (Poly19244) for mouse IHC was from Biolegend.

### Cell culture and conditions

The spontaneously immortalized human keratinocyte cell line (HaCaT) (Prof. Fusenig, Heidelberg, Germany) was cultured in DMEM (Invitrogen), 10% FCS (Biochrom), 1% penicillin/streptomycin solution (Invitrogen). NHK (normal human keratinocytes) cells were isolated from human juvenile foreskin and cultured in keratinocyte growth medium (Promo Cell) at 37°C in 5% CO_2_ atmosphere.

### Separation of keratinocyte populations

Early passage keratinocytes were divided in KSCs, TA and PM cells on the basis of their ability to adhere to type IV collagen, as described elsewhere [19]. Briefly, cells were allowed to adhere to type IV collagen dishes for 5 min (KSC), and non-adherent cells were transferred to fresh collagen-coated dishes and allowed to attach overnight (TA). Non-adherent cells belong to the PM population. Isolation of these cells was verified by the expression of β1 integrin and keratin 6 (S1 Fig).

### Differentiation of keratinocytes

To drive HaCaT cells into differentiation by post-confluent growth, increasing cell numbers (0.3 up to 6*10^5^ cells / 12 well) were seeded. After 24h the higher cell numbers were confluent and differentiation was initiated. If not indicated otherwise, cells were harvested after another 48h. In NHK cells differentiation was induced by the addition of 2mM CaCl_2_ for at least 48h.

### Proliferation of keratinocytes

Quantification of cell proliferation was determined by a colorimetric XTT assay (Roche) or by BrdU incorporation using a colorimetric cell proliferation ELISA (Roche). The assays were carried out according to the product instruction manual. For XTT assay, 2*10^4^ HaCaT cells were seed in 96well plates in triplicates and treated as indicated for the individual assays. XTT reagent was usually added after 48h of treatment and absorption was measured 4h later. For BrdU assays, 1*10^4^ cells were seeded in triplicates and after 24h treated as indicated for the individual assays. Cells were labeled over night with BrdU reagent and incorporation of BrdU was assessed the next morning.

### siRNA mediated knockdown

HaCaT cells were reverse-transfected with Stealth™siRNA directed against Akt, Raptor or mTOR and BLOCK-IT™ Negative Control (Invitrogen). Briefly, 100pmol siRNA duplexes and 5μl Lipofectamine^®^ 2000 were diluted separately in OptiMEM-I medium (Invitrogen), mixed and 6*10^5^ HaCaT cells were added. Cells were seeded in 12 well plates with increasing cell densities according to the requirements of the following assays. NHK cells were seeded at 3*10^5^ cells/12 well and the next day transfected with 15pmol *Silencer*^®^Select siRNA for Raptor, mTOR or control siRNA and 9 μl Lipofectamine^®^ RNAiMAX. After another 48h cells were stimulated with 2mM CaCl_2_ for another 48h.

### Western immunoblotting

Cells were lysed in RIPA lysis buffer (Cell Signaling Technology,), normalized, subjected to SDS–PAGE and blotted onto PVDF membranes. After blocking in 5% milk/TBS-T, membranes were probed with the indicated antibodies and visualized with HRP-conjugated secondary antibodies using ECL Substrate (Pierce). Western blots were quantified densiometrically using BioRad ImageLab software by dividing the signal intensity of the band of interest by the signal intensity of actin or tubulin bands. The results very normalized to the control and mean values of at least three independent experiments were calculated. Significant differences were determined by ANOVA and p-values are described in the figure legends. If no level of significance is given, differences were usually not significant.

### Analysis of differentiation markers via quantitative RT-PCR

Total RNA was isolated using NucleoSpin RNA isolation kit (Machery&Nagel), transcribed using SuperScriptIII First-Strand Synthesis Mix (Life Technologies) and subjected to qRT-PCR using predesigned TaqMan® Gene Expression Assay probes (ThermoFisher) on an AbiPrism 7500 Fast Sequence Detector. mRNA expression was normalized to RPLPO and relative changes in the respective mRNA were quantified by the 2^−ddCt^ method.

### Immunhistochemistry

20 psoriasis patients between 18–75 years with a confirmed diagnosis of severe plaque-type psoriasis vulgaris for at least 6 month and no current systemic anti-inflammatory therapy were recruited from the clinical research department of the dermatology department of the Clinic of the Goethe-University, Frankfurt, Germany. Five healthy individuals were recruited among employees of our clinic. Written informed consent was given and the study was approved by the ethics committee of the Clinic of the Goethe-University (144/12); the Declaration of Helsinki protocols were followed. Punch biopsies (6mm) from lesional skin of patients or normal skin of healthy individuals were taken. These were cut into 8 μm cryosections, fixed in methanol or acetone (Raptor) and permeabilized with TBS-T. Specimens were blocked with 5% goat serum/TBS-T and incubated overnight at 4°C with primary or isotype antibodies. After washing, samples were incubated with AlexaFluor488 labeled secondary antibody and nuclei were stained with DAPI. Confocal images were generated using a ZeissLSM510 microscope.

### Mice experiments

BALB/c mice (Charles-River) were housed in the animal facility of the Center for Medical Research, Medical University of Graz, Austria. All procedures were approved by the Austrian Government, Federal Ministry for Science and Research, (BMWF-66-010/0032-11/3b/2013) and conducted according to the NIH Guide for the Care and Use of Laboratory Animals. Mice (8–10 weeks) received MHY1485, dissolved in ethanol/propylene glycol in a ratio of 3:7 or vehicle alone topically once daily to the dorsal skin in a cumulative manner with increasing doses every third day (0.1 mg/ml, 0.3 mg/ml, 1 mg/ml, 3 mg/ml, 10 mg/ml). Double skin-fold thickness (DSFT) was assessed daily by measuring dorsal skin with a spring-loaded engineer’s micrometer (Mitutoyo). Mice were sacrificed 48h after the highest dose was applied. Mice were euthanized with an overdose of isoflurane and all efforts were made to minimize suffering. Blood serum was collected and stored at -80˚C for later use. Approximately 1 cm^2^ of central dorsal skin per mouse was excised, fixed immediately in 4% buffered formaldehyde, paraffin embedded and sectioned for H&E staining. Images were acquired by using a DP71 digital camera (Olympus, Melville, NY) attached to an Olympus BX51 microscope. Epidermal hyperplasia was monitored by counting epidermal cell layers at five randomly selected consecutive microscopic fields (at final magnification, x200). For quantification of epidermal thickness, five randomly selected measurements per H&E-stained cross-section of dorsal skin from each mouse were performed. All measurements were performed in a blinded manner. Results were first averaged per mouse and then averaged per treatment group for statistical analysis.

For immunhistochemistry staining paraffin sections were processed routinely. Primary antibody was applied overnight after pretreatment with Dako Retrieval solution of pH 6. Dako REAL Detection System (HRP/AEC, Rabbit/ Mouse) was used for detection, according to the manufacturer’s instructions. Images were acquired by using a DP71 digital camera (Olympus) and an Olympus BX51 microscope.

### Bead immunoassay

Mouse serum cytokine and chemokine levels were measured with Mouse Cytokine/Chemokine bead immunoassay kit, ProcartaPlex, 26Plex from Affymetrix eBioscience according to the manufacturer’s specifications using the Bio-Plex 20 (Bio-Rad) and analyzed with five parametric curve fitting.

## Results

### mTOR signaling is deactivated during keratinocyte differentiation

**Fig 1:**
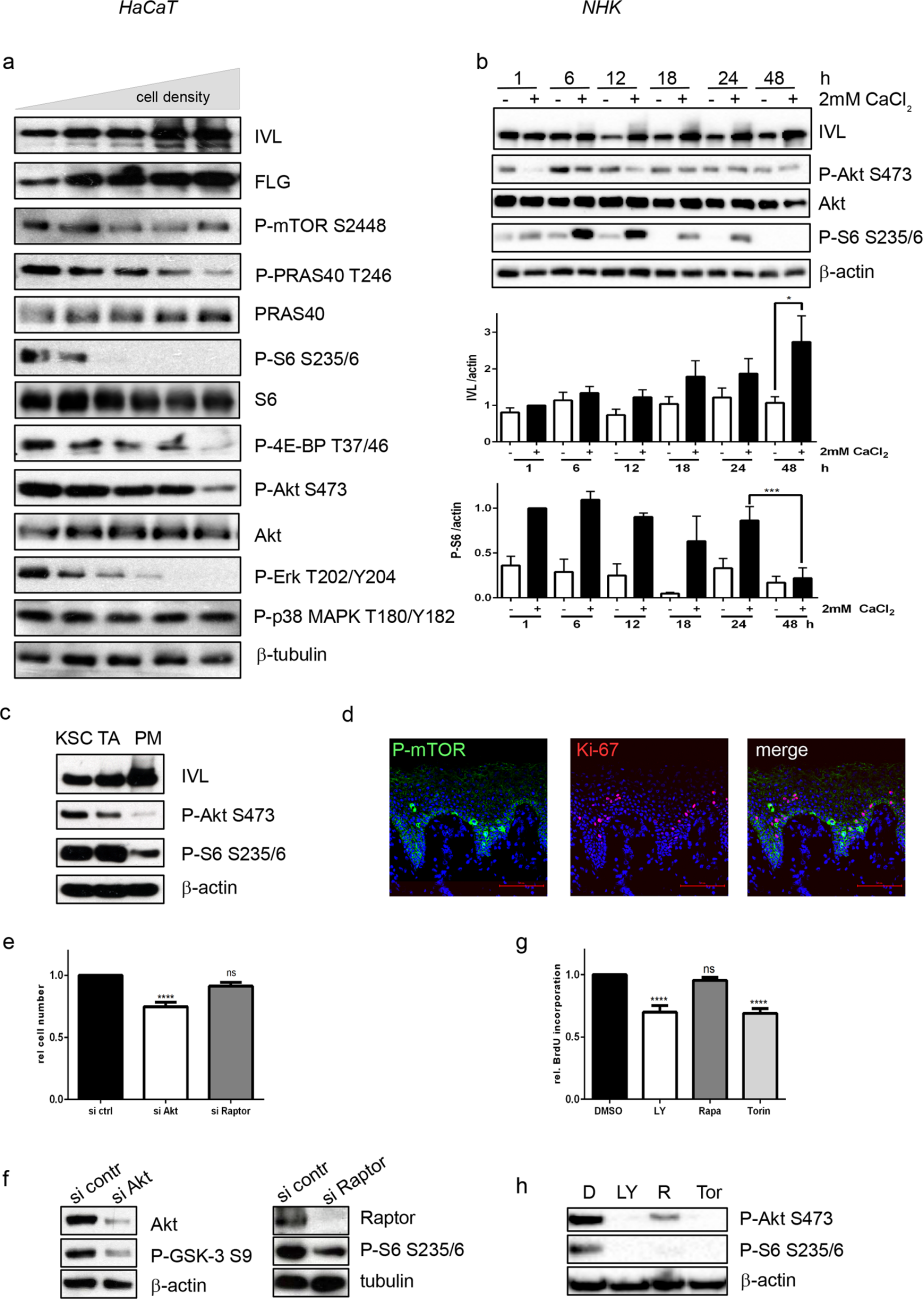
mTOR signaling is deactivated during differentiation and only partially contributes to the control of proliferation. (a) Increasing densities of HaCaT were seeded to promote differentiation and harvested after 72h. Protein lysates were subjected to SDS-PAGE and Western Blotting with the indicated antibodies. (b) NHK were serum-starved and differentiation was induced with 2mM CaCl_2_ for the indicated time points. Protein lysates were subjected to SDS-PAGE and Western Blotting with the indicated antibodies was performed. Below a densiometrical quantification of involucrin and P-S6 levels of n = 4–5 similar blots is shown. Statistical significance was calculated with one-way ANOVA and Bonferroni multiple comparison (* p ≤0.05, ***p ≤0.001). (c) Keratinocytes stem cells (KSC), transient amplifying (TA) and postmitotic (PM) cells were separated according to their ability to adhere to type IV collagen. Protein lysates were subjected to SDS-PAGE and Western blotting with the indicated antibodies, showing that mTORC1 signaling is mainly present in undifferentiated cells. (d) Normal human skin was stained with P-mTOR S2448 and Ki-67 antibody. Nuclei were stained with DAPI. Single color and overlay images are presented, which show that mTOR is activated in proliferation cells of the basal layer. Bars represent 100 μm. (e) HaCaT cells were reverse-transfected with siRNA targeting Akt, Raptor or control siRNA and seeded in 96 well plates. After 48h proliferation was quantified using the XTT-based assay. Graph presents mean ± SEM (n = 4–8). Statistical significance was calculated with one-way ANOVA and Bonferroni multiple comparison (****p ≤0.0001, ns: non-significant). (f) To control for the efficiency of knockdown, HaCaT cells were transfected as in (e) and seeded in 6 well plates. Protein lysates were harvested after 48h and a Western blot was performed with the indicated antibodies. To show the consequences of Akt and Raptor knockdown, phosphorylation of the downstream targets GSK-3 and S6 was also captured. (g) HaCaT cells were seeded in 96 well-plates in triplicates and after 24h 50 μM LY294002, 100 nM Rapamycin or 250 nM Torin or solvent (DMSO) were added. After another 48h cell proliferation was measured with a BrdU assay. Graph presents mean ± SEM (n = 6). Statistical significance was calculated with one-way ANOVA and Bonferroni multiple comparison (****p ≤0.0001, ns: non-significant). (h) To show efficiency of the used inhibitors, HaCatT cells were treated as in (g). Protein lysates were prepared and a Western blot was performed with the indicated antibodies.

To analyze the contribution of mTOR signaling to keratinocyte maturation, we used two different models of keratinocyte differentiation. HaCaT cells were driven into differentiation by post-confluent growth and differentiation was measured by the expression of involucrin and filaggrin. At the same time the mTOR pathway was shut off as measured by the phosphorylation of the mTOR kinase itself, PRAS40, 4E-BP1 and the ribosomal protein S6 (Fig 1A). However, other proliferative pathways such as Akt or Erk1 were also turned off. This was not a general phenomenon, as p38MAPK remained active (Fig 1A). In NHK (normal human keratinocytes) differentiation was induced by the addition of 2mM CaCl_2_, which led to the expression of involucrin beginning between 12 and 18h resulting into a strongly differentiated state at later time points. (Fig 1B). This was also seen on the RNA level by the induction of differentiation markers of different maturation stages (keratin1, involucrin, loricrin, transglutaminase and filaggrin) within a similar period (S1 Fig). Interestingly Ca^2+^ initially activated mTOR signaling as measured by phosphorylation of S6, but as cells became further differentiated, starting at 18h, activation of Akt and S6 started to decline (Fig 1B). Thus we hypothesize that deactivation of mTOR signaling is important for progression of keratinocyte differentiation.

### mTOR signaling plays a minor role in regulating keratinocyte proliferation

To verify whether the shutdown of mTOR signaling is part of the differentiation process in the normal epidermis, we separated keratinocyte stem cells (KSC), transient amplifying (TA) and post-mitotic (PM) cells from primary human keratinocytes. While KSCs of the basal epidermal layer expressed low amounts of the differentiation marker involucrin, they showed high mTOR activity as measured by S6 phosphorylation (Fig 1C, S2 Fig). TA cells, that are about to leave the basal layer for differentiation, started to downregulate PI3-K signaling as measured by reduced Akt phosphorylation. In contrast, PM cells that are determined for terminal differentiation completely shut down their mTOR signaling and only little S6 phosphorylation could be detected in these cells (Fig 1C, S2 Fig). As these cells with high mTOR activity (KSC and TA cells) only represent a small proportion of the epidermis and we did not see active mTOR signaling in healthy skin before [17], we used a different staining protocol. We found that mTOR was active in certain cells of the basal layer and that some of these cells were also positive for Ki-67 (Fig 1D). Hence, we asked whether mTOR plays a role in regulating cell proliferation. Blocking Akt, as an upstream regulator of mTORC1 activity with either siRNA knockdown or LY294002 (Fig 1F abd 1H), impeded keratinocyte proliferation (Fig 1E and 1G). However, inhibition of mTORC1 signaling with rapamycin or Raptor knockdown (Fig 1F and 1H) had hardly any effect on keratinocyte proliferation (Fig 1E and 1G, S3 Fig). Noteworthy blocking both mTOR complexes with Torin1 and thus blocking Akt (Fig 1H) had a strong inhibitory effect on proliferation (Fig 1G). This argues that Akt regulates keratinocyte proliferation mainly via other pathways, while mTOR might have other functions in regulating the switch from proliferation to differentiation.

### Inactivation of mTOR signaling is important for keratinocyte differentiation

**Fig 2:**
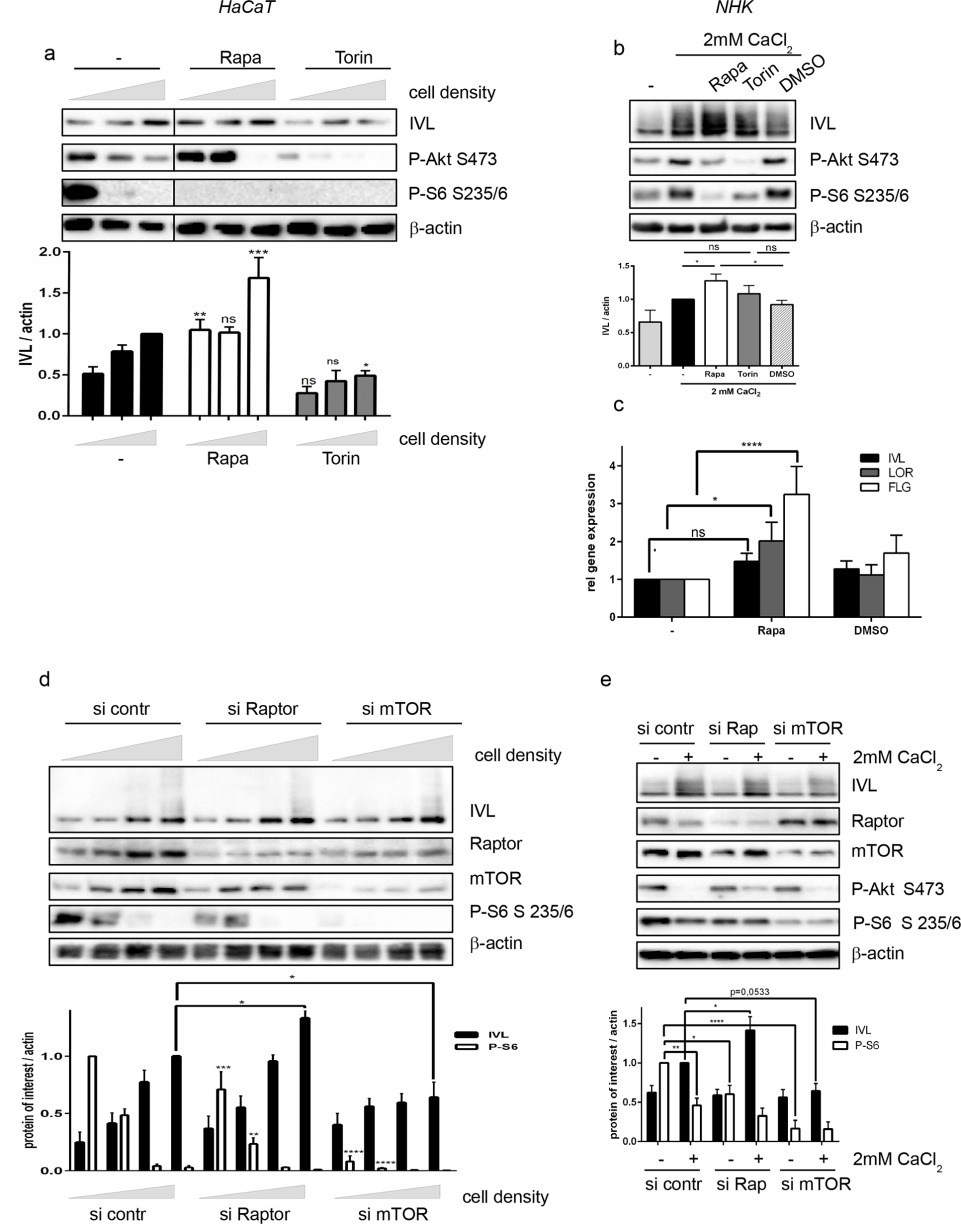
Inhibition of mTORC1 signaling promotes differentiation. (a) Increasing cell numbers of HaCaT cells were seeded and after 24h 100 nM Rapamycin, 250 nM Torin or solvent control (DMSO) were added and differentiation was allowed to proceed for 72h. Protein lysates were subjected to Western blotting and proteins were detected with the indicated antibodies. Quantification of 3–6 similar blots is depicted below. Statistical significance was calculated with two-way ANOVA and Bonferroni multiple comparison, comparing different treatments with the corresponding cell density in the control group (*p ≤0.05, ***p ≤0,001, ns: non-significant). (b) In NHK cells differentiation was induced with 2mM CaCl_2_ in the presence of 100nM Rapamycin, 250 nM Torin or solvent control for 48h. Protein lysates were subjected to Western blotting and proteins were detected with the indicated antibodies. Below a quantification of six similar blots is shown. Statistical significance was calculated with one-way ANOVA and Bonferroni multiple comparison (*p ≤0.05). (c) NHK cells were treated with 2 mM CaCl_2_ and 100nM Rapamycin or DMSO control. RNA was isolated and quantitative RT-PCR was performed to measure expression of the indicated differentiation markers. Graph presents mean ± SEM (n = 5–8). Statistical significance was calculated with two-way ANOVA and Bonferroni multiple comparison. (*p ≤0.05, ****p ≤0.0001, ns: non-significant). (d+e) HaCaT (d) or NHK (e) cells were transfected with siRNA specific for Raptor (Rap), mTOR or an siRNA control (si contr) and differentiation was induced either by post-confluent growth (d) or with 2mM CaCl_2_ (e) for 48h. Protein lysates were analyzed by Western blotting with the indicated antibodies. Below each blot a quantification of IVL and P-S6 band relative to actin bands of 4–6 similar blots is shown. Statistical significance was calculated with 2-way ANOVA and Bonferroni multiple comparison, comparing Raptor or mTOR knockdown with the corresponding differentiation state in the control group (*p ≤0.05, ** p ≤0,01, ****p ≤0,0001).

To investigate this, mTORC1 signaling was inhibited in both cellular differentiation models. Rapamycin treatment enhanced the expression of differentiation markers: The expression of involucrin was increased on the protein level (Fig 2A and 2B), and enhanced expression of the mRNA of more differentiation markers such as involucrin, loricrin and filaggrin was detected (Fig 2C). Similar results were obtained when mTORC1 signaling was inhibited by siRNA knockdown of Raptor (Fig 2D and 2E). The expression of involucrin was increased in Raptor-deficient cells compared to control cells at all stages of differentiation, however a significant increase could be only measured for the highest cell density. Thus, we assume that mTOR signaling has to be below a critical level for differentiation to progress. In Raptor deficient cells, this can be achieved at an earlier time point than in control cells. However, blocking the mTOR kinase itself using either Torin or mTOR siRNA had the opposite effect by reducing the expression of involucrin (Fig 2A, 2B, 2D and 2E), which we assume is due to the role of mTORC2 in phosphorylating Akt, as Akt knock-down also impeded differentiation (S4 Fig). Thus, we argue that inactivation of mTORC1 signaling is a prerequisite for the progression of differentiation.

### mTOR signaling is highly active in lesional psoriatic skin

**Fig 3:**
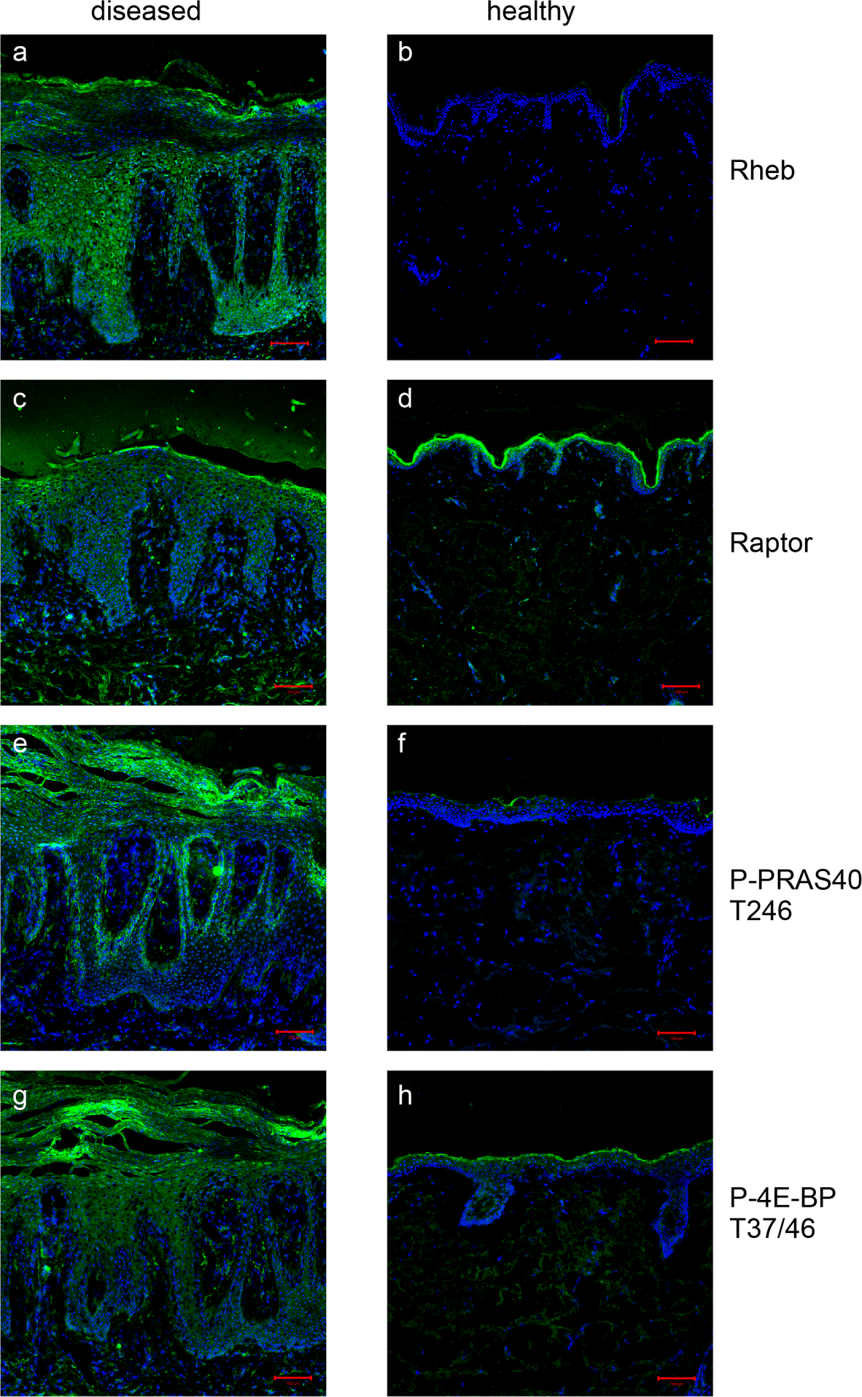
mTORC1 and its downstream mediator are strongly activated in psoriatic lesions. Punch biopsies from lesional psoriatic skin (a,c,e,g) of 20 patients and five healthy donors (b,d,f,h) were stained with antibodies for specific for Rheb, Raptor, P-PRAS40 and P-4E-BP. Nuclei were stained with DAPI. Representative overlay images from one patient and one healthy donor are shown. Bars represent 100 μm.

In psoriasis, the balance between proliferation and differentiation is strongly disturbed. To further investigate the role of mTOR signaling in psoriasis, lesional skin was stained for additional components of the mTOR complex and downstream signaling molecules. Rheb (Fig 3A) and Raptor (Fig 3C) were strongly overexpressed in lesional psoriatic skin compared to healthy skin (Fig 3B and 3D). PRAS40 showed a similar activation pattern as the mTOR kinase itself: activation over the whole epidermis of lesional skin but especially intense phosphorylation in the stratum basale (Fig 3E) and hardly any activity in healthy skin (Fig 3F). 4E-BP1 was also strongly activated in lesional skin (Fig 3G), compared to control skin (Fig 3H). In summary, hyperactivation of different components of mTOR signaling was detected in psoriatic skin with differential localization over the epidermis.

### Proinflammatory psoriatic cytokines activate the mTOR pathway via PI3-K signaling

**Fig 4:**
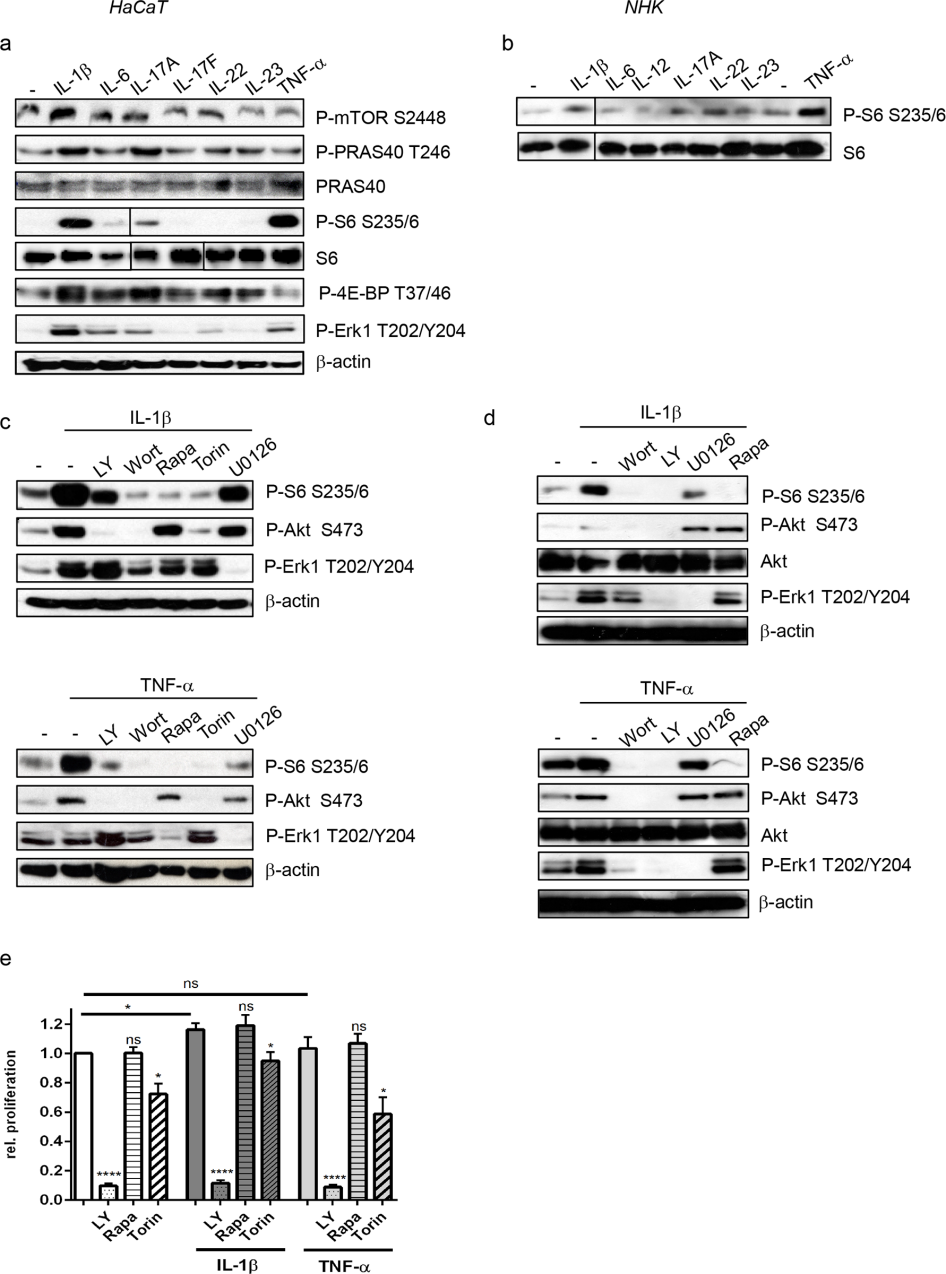
Psoriatic cytokines induce mTOR signaling. Starved HaCaT (a) or NHK cells (b) were treated for 30 min with 20 ng/ml of the indicated cytokines. Serum starved HaCaT (c) or NHK cells (d) were treated for 30 min with the indicated inhibitors (1 μM Wortmannin, 50 μM LY294002, 50 μM U0126 or 100 nM Rapamycin), followed by a 30 min stimulation with IL-1 β or TNF- α (20ng/ml). Cell lysates were analyzed by Western blotting with antibodies for the indicated proteins. (e) HaCaT cells were seeded in triplicates in 96 well plates, starved overnight and treated with 50 μM LY 294002, 100 nM Rapamycin or 250 nM Torin or solvent (DMSO) as well as 20 ng/ml IL-1 β or TNF- α. After 48h cell proliferation was measured with a BrdU assay. Graph presents mean ± SEM (n = 6). Statistical significance was calculated with one-way ANOVA and Bonferroni multiple comparison (*p ≤0.05, ****p ≤0.0001). This shows that mTORC1 does not play a major role in controlling keratinocyte proliferation.

When examining the mediators, that could be responsible for this strong activation of mTOR signaling, we found that IL-1β, IL-17A and TNF-α strongly activated the mTOR kinase, PRAS40 and the downstream targets of mTOR activity 4E-BP1 and the ribosomal protein S6 (Fig 4A and 4B). IL-6 and IL-22 only mediated mild activation of the mTOR pathway, while IL-17F and IL-23 did not show any effect (Fig 4A and 4B). Using PI-3K inhibitors showed that IL-1β and TNF-α dependent activation of mTOR signaling was mediated via PI3-K (Fig 4C and 4D). IL-1β, IL-17A and TNF-α were also able to activate MAPK/Erk1 signaling (Fig 4A), which only partially contributed to cytokine-dependent mTORC1 activation signaling in keratinocytes as MEK inhibition with U0126 did not fully prevent cytokine dependent activation of mTOR (Fig 4C and 4D).

Although mTOR signaling did not regulate keratinocyte proliferation under normal conditions, IL-1β as an mTOR activator induced cell proliferation to some degree, while TNF-α only had a small but not significant effect on proliferation (Fig 4E). However, this effect was not mediated via mTOR as rapamycin did not block proliferation. In contrast, inhibition of PI3-K with LY294002 strongly blocked proliferation. Interestingly, blocking both mTOR complexes with Torin and thereby also blocking Akt had a smaller inhibitory effect on proliferation than seen under basal conditions (Fig 1G).

In summary this data suggest that also under inflammatory conditions keratinocyte proliferation is mainly regulated via Akt and that mTOR signaling only partially contributes to this process.

### Cytokine mediated induction of mTOR interferes with proper keratinocyte differentiation

**Fig 5:**
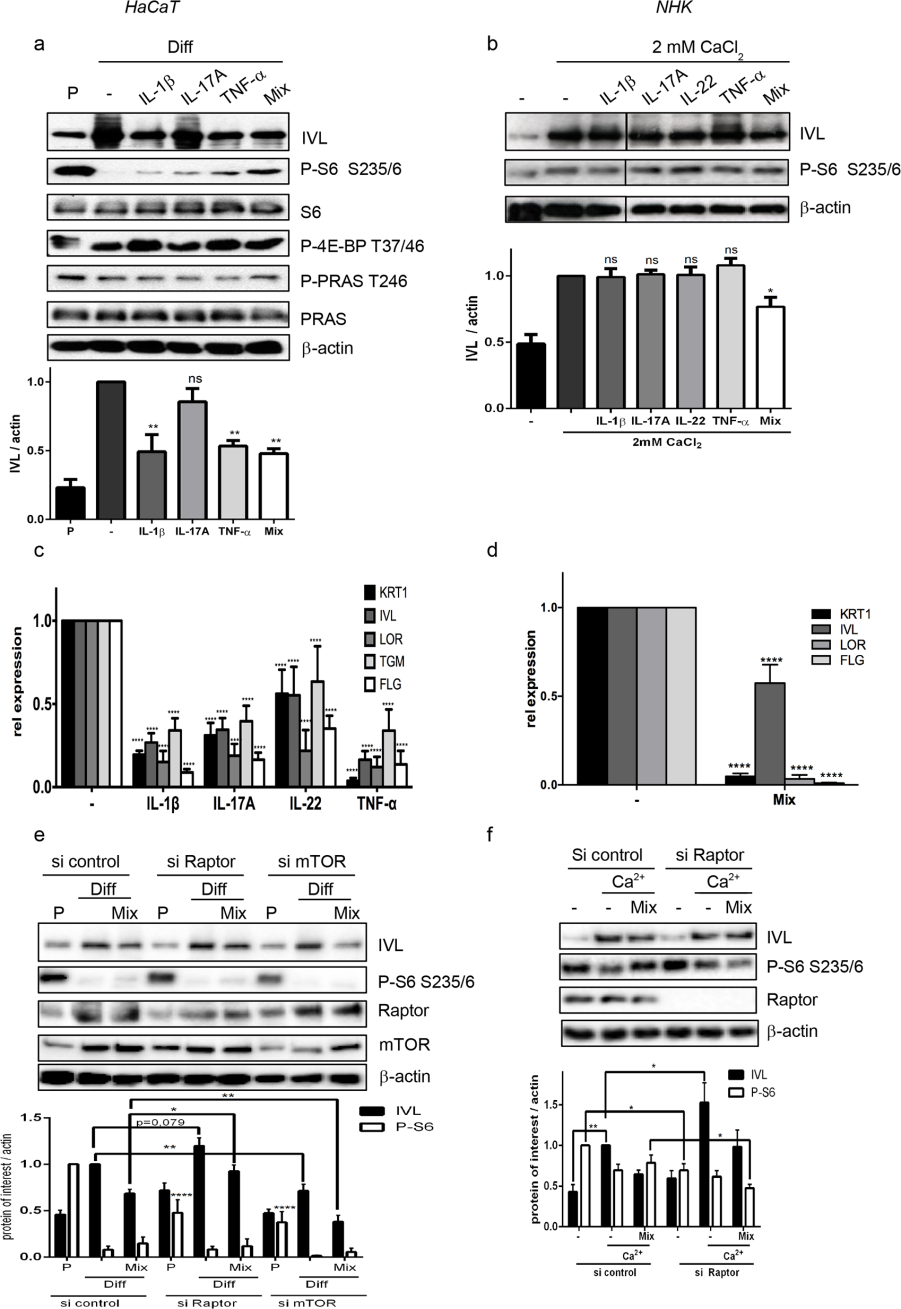
Cytokine mediated activation of mTOR interferes with differentiation. (a,c) HaCaT cells were seeded at proliferating (P; 0,6 *10^5^ cells/12 well) or differentiating (Diff; 6* 10^5^ cells/12 well) conditions and treated with 20 ng/ml of the indicated cytokines or a mix of IL-1 β, Il-17A and TNF- α. (b,d) NHK cells were treated with 2mM CaCl_2_ to induce differentiation and 20 ng/ml of the indicated cytokines or the mix of these cytokines. After 72h protein and RNA were isolated. (a,b) Protein samples were analyzed by Western blotting with the indicated antibodies. Below each blot quantification of n≥ 3 similar Western blots is depicted. Statistical significance was calculated with one-way ANOVA and Bonferroni multiple comparison (*p ≤0.05, **p ≤0.01). (c,d) Quantitative RT-PCR was performed to measure expression of the indicated differentiation markers. Graphs present mean ± SEM (n*≥*5). Statistical significance was calculated with two-way ANOVA and Bonferroni multiple comparison. (****p ≤0.0001). (e) HaCaT cells were reverse-transfected with siRNA specific for Raptor, mTOR or control siRNA and seeded at 0,6 *10^5^ (P) or 6*10^5^ (Diff) cells per 12 well. After 24h cells were treated with 20 ng of IL-1 β, IL-17A and TNF- α (Mix) and harvested after another 72h. Protein lysates were analyzed by Western blotting with the indicated antibodies. Below a quantification of 7–9 similar blots is shown. Statistical significance was calculated with two-way ANOVA and Bonferroni multiple comparison. For P-S6 statistical significant difference is shown for proliferating cells of knockdown cells compared to proliferating control cells (*p ≤0.05, **p ≤0.01, **** p ≤0.0001). (f) NHK cells were transfected with siRNA specific for Raptor or control siRNA. After 24h the cytokine mix and 2 mM CaCl_2_ were added. Differentiation was allowed to proceed for 48h, cells were harvested and protein lysates were analyzed by Western blotting with the indicated antibodies. Below a quantification of seven similar blots is shown. Statistical significance was calculated with two-way ANOVA and Bonferroni multiple comparison (*p ≤0.05, **p ≤0.01). Raptor knockdown rescues the cytokine induced repression of keratinocyte differentiation.

As low mTORC1 signaling seems to be favorable for proper differentiation, we asked whether aberrant activation of mTORC1 by inflammatory cytokines is the reason for the differentiation defect in the psoriatic epidermis. Treating differentiating HaCaT cells with IL-1β, TNF-α and a mix of IL-1 β, TNF- α and IL-17A repressed the expression of involucrin on the protein (Fig 5A) and of keratin1, involucrin, loricrin and filaggrin on the mRNA level (Fig 5C), which was in parallel with their capacity to activate mTOR signaling (Fig 5A). In contrast, IL-17A and IL-22, which only showed mild activation of mTORC1 did not interfere with the expression of involucrin (Fig 5A). In NHK, the effect of the single cytokines on differentiation was not that prominent (Fig 5B and S5 Fig), however especially the mix was able to strongly repress the expression of all differentiation markers (Fig 5B and 5D). To verify that mTOR signaling is functionally involved in mediating this effect, we knocked out Raptor using siRNA, which specifically blocks mTORC1 signaling. Raptor knockdown not only induced upregulation of involucrin under differentiating condition as also seen in Fig 2D and 2E, but was able to partially rescue cytokine-induced repression of differentiation as measured by the expression of involucrin (Fig 5E and 5F). At least in HaCaT cells this rescue was significant when comparing Raptor knockdown cells with control cells (p ≤0.05) (Fig 5E). In contrast, we could only find a trend in NHK cells (Fig 5F), which believe is due to the greater experimental variance in these primary cells. By calculating the relative repression by the mix compared to differentiating cells in HaCaT (0.68 in si control vs 0.77 in si Raptor) we verified that there was indeed a trend towards normalization of involucrin expression in raptor knockdown cells and could exclude that this effect was only due to the general induction of involucrin by raptor knockdown. In contrast when both mTOR complexes were inhibited through knockdown of the mTOR kinase itself, the level of differentiation was lower as seen before and no rescue of cytokine induced differentiation repression could be detected (Fig 5E). Thus, we assume that psoriatic cytokines induce strong mTORC1 activity in the epidermis, which prevents proper epidermal stratification.

### Hyperactivation of mTORC1 signaling by MHY1485 induces a psoriasis-like skin morphology

**Fig 6:**
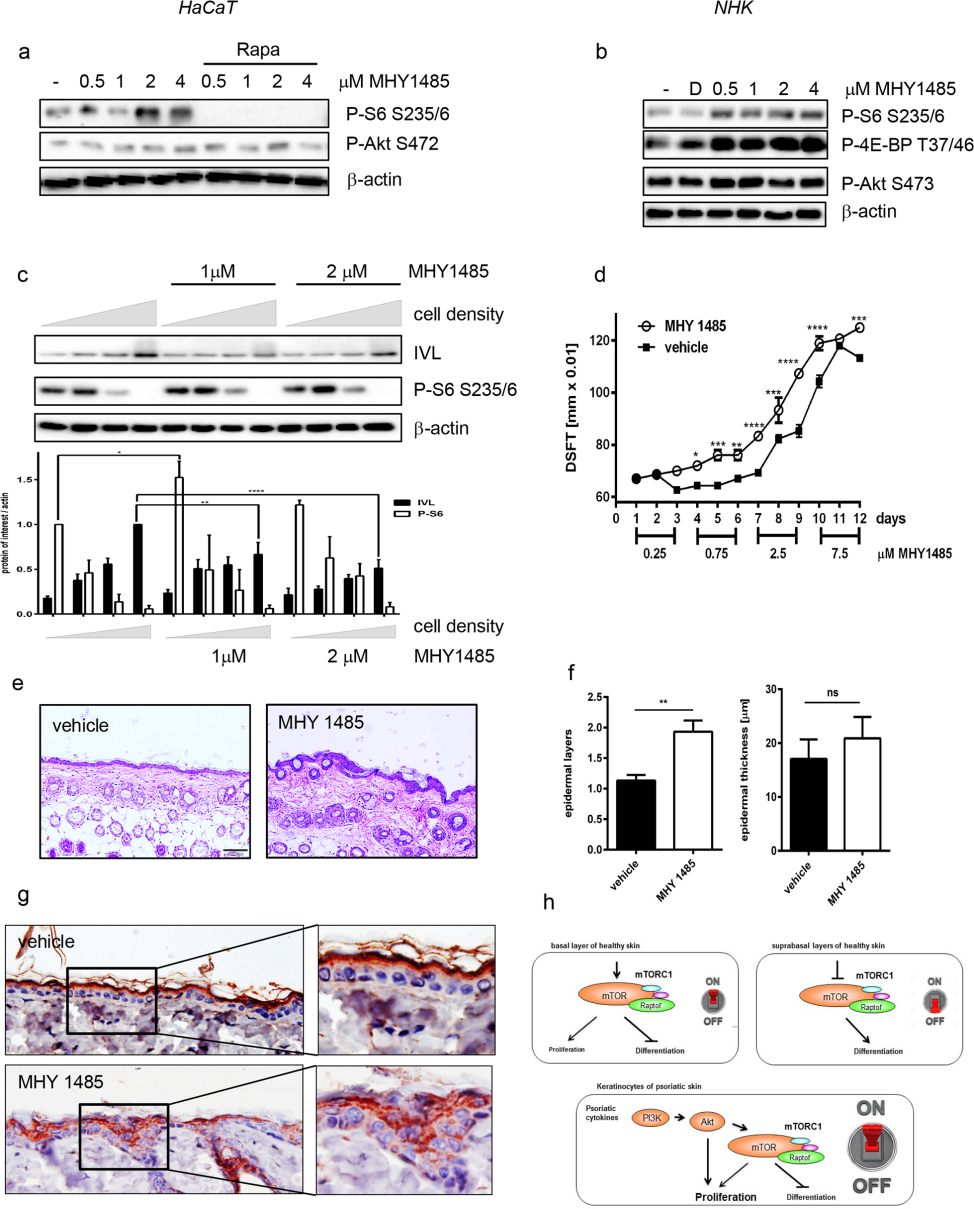
Activation of mTORC1 signaling inhibits differentiation. HaCaT cells (a) or NHK cells (b) were serum starved overnight and treated with increasing doses of MHY1485 or DMSO for 30 min If indicated cells were pre-treated with 100 nM Rapamycin for 30 min. Cells were harvested and protein lysates were analyzed by Western blotting with the indicated antibodies, showing that MHY1485 induces mTORC1 signaling. (c) Increasing numbers of HaCaT cells were seeded and driven into differentiation by post-confluent growth in the presence of the indicated concentrations of MHY1485. Protein lysates were analyzed by Western blotting with the indicated antibodies. Below a quantification of 6–8 similar Western blots is shown. Statistical significance was calculated with two-way ANOVA and Bonferroni multiple comparison (*p ≤0.05, **p ≤0.01). (d-g) MHY1485 or vehicle control was topically applied to the dorsal skin of mice for 12 consecutive days with increasing doses. (d) DSFT (Double Skin Fold Thickness) was measured before the first treatment (day1) and repeated every day. Data shown are mean values from one experiment, with n = 3 mice per treatment. Statistical significance was calculated with two-way ANOVA and Bonferroni multiple comparison (*p ≤0.05, **p ≤0.01, *** p ≤0.001, **** p ≤0.0001). (e) Representative images of H&E-stained sections from dorsal skin of a mouse of control and MHY 1485 treated groups (scale bar, 100 μM). (f) Evaluation of histological features, including number of epidermal layers and epidermal thickness in μM. Data shown are mean values of five measurements per mouse ± SEM. Statistical significance was calculated with Mann-Whitney test (**p ≤0.01). (g) Involucrin staining of vehicle control or MHY1485 treated mice. Overview images and close-ups are shown. MHY1485 induces in vivo a psoriasis like phenotype and interferes with proper differentiation (h) Hypothetical model how mTOR serves as a switch to determine the fate of keratinocytes.

To further verify that hyperactivation of mTORC1 is the reason for improper differentiation in psoriasis, we used the synthesized compound MHY1485 that was designed to specifically activate mTOR signaling [20]. MHY1485 induced mTORC1 signaling as shown by the phosphorylation of S6 or 4E-BP1 (Fig 6A and 6B), which was mediated by mTORC1 as pretreatment with rapamycin completely blocked the effect of MHY1485 (Fig 6A). mTORC2 signaling is not influenced as the phosphorylation of Akt at S473 remains unaltered (Fig 6A and 6B). Applying MHY1485 to the HaCaT differentiation model maintained continuous mTORC1 signaling; especially a significant increase in S6 phosphorylation could be detected during early differentiation. In untreated control cells S6 phosphorylation faded much earlier during differentiation. The continuous mTORC1 activation by the agonist impeded differentiation as measured by significantly reduced involucrin expression especially during later differentiation. However, the effect of the agonist was not strong enough to maintain S6 phosphorylation under fully differentiated conditions, resulting in residual expression of involucrin at the highest cell density (Fig 6C).

To substantiate these findings *in vivo*, MHY1485 was applied to the dorsal skin of mice with increasing doses. While the skin looked macroscopically nearly normal, the dorsal skin fold thickness (DSFT) was increased over the course of the experiment in MHY1485 treated animals (Fig 6D). Histological analysis revealed a psoriasis-like morphology (Fig 6E) characterized by more epidermal layers and a small but not significant acanthosis (Fig 6F). At the same time, proper differentiation was disturbed as involucrin was delocalized and also expressed in the spinous and granular layer, while in control mice it was exclusively detected in the upper granular and corneal layer (Fig 6G). However, topical application of MHY1485 did not lead to a systemic effect that resembled the situation in psoriasis as no induction of chemokines and Th1/Th17 cytokines could be detected (S6 Fig).

In summary, our data showed that in healthy skin, deactivation of mTORC1 signaling seems to be required for basal keratinocytes to progress through the epidermal maturation process. In contrast, in psoriasis overexpression of Th17 cytokines induces strong activation of mTOR signaling which prevents keratinocytes from proper differentiation in order to form the correctly stratified epidermis.

## Discussion

We could show that healthy skin keratinocyte stem cells of the basal layer display high PI3-K/ mTOR activity, that correlates with Ki-67 staining and thus with their proliferative activity. This is supported by a recent study by Ding *et al*. that shows that epidermal loss of mTORC1 signaling results in reduced proliferation of epidermal progenitors cells in the basal layer leading to a hypoplastic epidermis during development [21]. Interestingly, we could not see a strong effect of mTOR inhibition on proliferation *in vitro*, which can be explained with the fact that cultivated keratinocytes represent a mixed population with a large proportion of cells, that have already left the cell cycle and are committed to differentiation.

The activity of mTOR greatly subsides when cells leave the basal layer and initiate differentiation. This seems to be necessary for proper keratinocyte maturation as blockade of mTORC1 facilitates the progression of differentiation. A possible mechanism could be the inhibition of autophagy by the mTOR complex [22]. Upon autophagic induction, phosphorylation of mTOR disappears, which is paralleled by differentiation [23]. Furthermore, differentiating keratinocytes undergo a selective form of nucleophagy, and mTORC1 signaling is critically involved in this process [24]. Interestingly, knockdown of the mTOR kinase blocked differentiation, which we assume is due to the effect of mTORC2 on Akt as knockdown of Akt also inhibited the expression of differentiation markers. This is in line with results by us [10] and others [25, 26] showing that Akt is active in the upper granular layer of health skin, where it likely contributes to nuclear degradation, which is an important step in the formation of the cornified envelope [27].

In contrast under inflammatory conditions such as in psoriasis, the vast presence of inflammatory cytokines of the TH1/17 family, constitutively activates mTORC1 signaling via PI3-K/ Akt, which can not only be seen in human psoriasis [17, 28] but also in different psoriasis mouse models and PUVA treatment significantly inactivated the mTOR pathway *in vitro* and *in vivo* [29, 30]. Interestingly, IL-22 is not only able to activate mTOR signaling but also induces the expression of mTOR mRNA [31]. Strong activation of mTORC1 in the basal compartment seems to be at least partially involved in proliferative control. This is underlined by reports that rapamycin blocks the proliferation of psoriatic keratinocytes [32].

However, the activity of mTORC1 in suprabasal layers of psoriatic skin could be indicative of a function of mTORC1 in aberrant maturation of the psoriatic epidermis. Cytokine dependent activation of mTOR signaling blocks differentiation, but can be rescued by mTORC1-specific inhibition. Keratin 6, which is upregulated within the psoriatic plaque and characteristic of hyperproliferative, cells in suprabasal layers [33] shows putative 5´TOP elements in its mRNA, which are specifically regulated by mTORC1. In addition, its expression is sensitive to rapamycin [34]. Thus, mTOR might regulate aberrant expression of keratin 6, which contributes to proliferation-associated keratinization and disturbs epidermal maturation. Another conceivable mechanism could be phosphorylation of STAT3 by mTOR [35], in the psoriatic epidermis [5]. STAT3 knock-in mice show mild epidermal hyperplasia coupled to aberrant keratinocyte differentiation with reduced expression of loricrin and filaggrin in suprabasal layers. Persistently elevated STAT3 signaling blocks keratinocyte differentiation *in vitro* [36] and *in vivo* probably by enforcing their stay in the proliferative compartment, which could be due to STAT3-dependent activation of proliferative genes such as cyclin D1 and c-Myc [37]. Moreover, a disturbed profile of autophagy expression markers could be seen in psoriatic skin, which suggests that aberrant mTOR activation in the inflamed skin inhibits proper autophagy needed for terminal differentiation [24].

We modeled this aberrant mTOR activation using the synthetic mTOR agonist MHY 1485, which induced psoriasis-like skin changes especially skin thickening and delocalization of involucrin. While involucrin is mainly localized in the granular layer in healthy human skin, it is overexpressed and mislocalized into the stratum spinosum in lesional psoriatic and therefor a sign for disturbed differentiation [38]. We assume that mTOR hyperactivation might have a dual effect on the processes leading to the psoriatic phenotype: In cells that still have the capacity to divide, mTORC1 signaling enforces proliferation contributing to the acanthosis seen in MHY1485 mice and in psoriasis. While in cells that are already determined for differentiation, the regular maturation program is blocked leading to aberrant epidermal maturation such as dislocation of involucrin.

Thus, mTOR inhibition seems a promising anti-psoriatic strategy. Remarkably, systemic rapamycin or its derivatives have been used for its immunosuppresive properties in anti-psoriatic trials alone or in combination with sub-therapeutic doses of cyclosporine and showed promising success [39–41]. However, our data support the notion that psoriasis patients could rather benefit from the topical use of mTOR inhibitors on the affected skin, which showed encouraging results in a small trial [42]. Locally applied it could not only inhibit the proliferation of psoriatic keratinocytes but also restore the epidermal differentiation defect [43].

In summary, we propose a model, where mTORC signaling serves as a switch between keratinocyte proliferation and differentiation (Fig 6H). In keratinocytes of the basal layer mTOR signaling is active and contributes to the control of proliferation while preventing differentiation. When cells leave the proliferative compartment, mTOR signaling is switched off which promotes differentiation. However, under inflammatory conditions this switch is hijacked by inflammatory cytokines, which prevents proper differentiation while promoting massive proliferation via Akt, leading to the phenotypic changes as seen in psoriasis. Thus controlling mTOR signaling might be a useful strategy to restore proper stratification of the psoriatic epidermis.

## Supporting information

S1 Fig Ca^2+^ dependent expression of differentiation markers over time. NHK were serum-starved and differentiation was induced with 2mM CaCl_2_ for the indicated time points. RNA was isolated and quantitative RT-PCR was performed to measure expression of the indicated differentiation markers. Graph presents mean ± SEM (n = 3–7). Statistical significant difference between Ca^2+^ treated and control for each time point was calculated with one-way ANOVA and Bonferroni multiple comparison (***p≤0.001, ****p ≤0.0001).
(TIF)

S2 Fig mTOR signaling is switched off when keratinocytes mature. Keratinocytes stem cells (KSC), transient amplifying (TA) and postmitotic (PM) cells were separated according to their ability to adhere to type IV collagen. In addition NHK were seeded in a normal cell culture dish without further separation (all). Protein lysates were subjected to SDS-PAGE and Western blotting with the indicated antibodies.
(TIF)

S3 Fig mTORC1 does not play a major in the proliferative control of keratinocytes. HaCaT cells were reverse-transfected with siRNA targeting Raptor or control siRNA and seeded in 96 well plates. After 72h proliferation was quantified using a BrdU assay. Graph presents mean ± SEM (n = 6).
(TIF)

S4 Fig Akt Knockdown blocks differentiation. HaCaT cells were reverse-transfected with siRNA specific for Akt or a siRNA control (si contr) and differentiation was induced by post-confluent growth for 72h. Protein lysates were analyzed by Western blotting with the indicated antibodies. Below each blot a quantification of n≥ 3 similar blots is shown. Statistical significant differences between control and knockdown cells of the same density were calculated with one-way ANOVA and Bonferroni multiple comparison (****p ≤0.0001).
(TIF)

S5 Fig Single cytokines are not able to interfere strongly with differentiation in NHK cells. NHK cells were seeded and 24h later, differentiation was induced by the addition of 2 mM CaCl_2_ in the presence of 20 ng/ml of IL-1β, IL-17A, IL-22 or TNF- α or a mix of IL-1 β, IL-17A and TNF- α. After 72h RNA was isolated and quantitative RT-PCR was performed to measure expression of the indicated differentiation markers. Graph present mean ± SEM (n = 4–8). Statistical significant difference between Ca^2+^ and the cytokines was calculated with one-way ANOVA and Bonferroni multiple comparison (*p≤ 0.05, **p≤0.01, ****p ≤0.0001).
(TIF)

S6 Fig Topical application of MHY does not influence serum cytokine levels. Mice were treated, as described in Fig 6D. At the end of treatment regimen, serum samples were collected and analyzed for protein expression of 26 cytokines and chemokines using multiplex bead immunoassay. IL-17, IL-23, IL-12, IL-1 β, IL-10, IL-6 and GM-CSF levels were not detectable. Data shown are from one experiment, with n = 2–3 mice per treatment group.
(TIF)
