# Supplementary figures and images for: Inflammation dependent mTORC1 signaling interferes with the switch from keratinocyte proliferation to differentiation
Source: PLoS One. 2017 Jul 10;12(7):e0180853. doi: 10.1371/journal.pone.0180853 (PMC5507280; doi:10.1371/journal.pone.0180853)

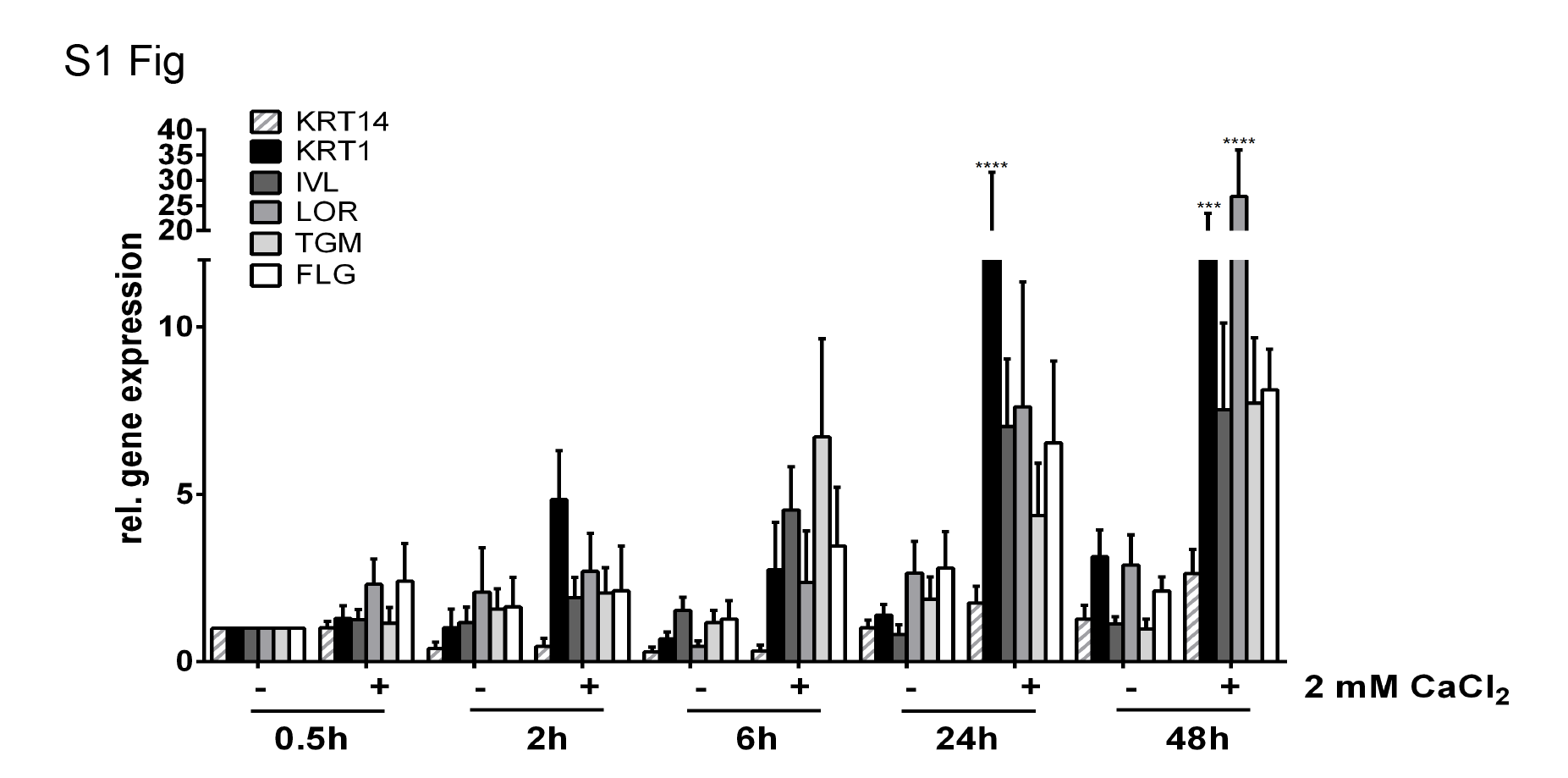

Supplement: S1 Fig — NHK were serum-starved and differentiation was induced with 2mM CaCl2 for the indicated time points. RNA was isolated and quantitative RT-PCR was performed to measure expression of the indicated differentiation markers. Graph presents mean ± SEM (n = 3–7). Statistical significant difference between Ca2+ treated and control for each time point was calculated with one-way ANOVA and Bonferroni multiple comparison (***p≤0.001, ****p ≤0.0001). (TIF) [file pone.0180853.s001.tif]

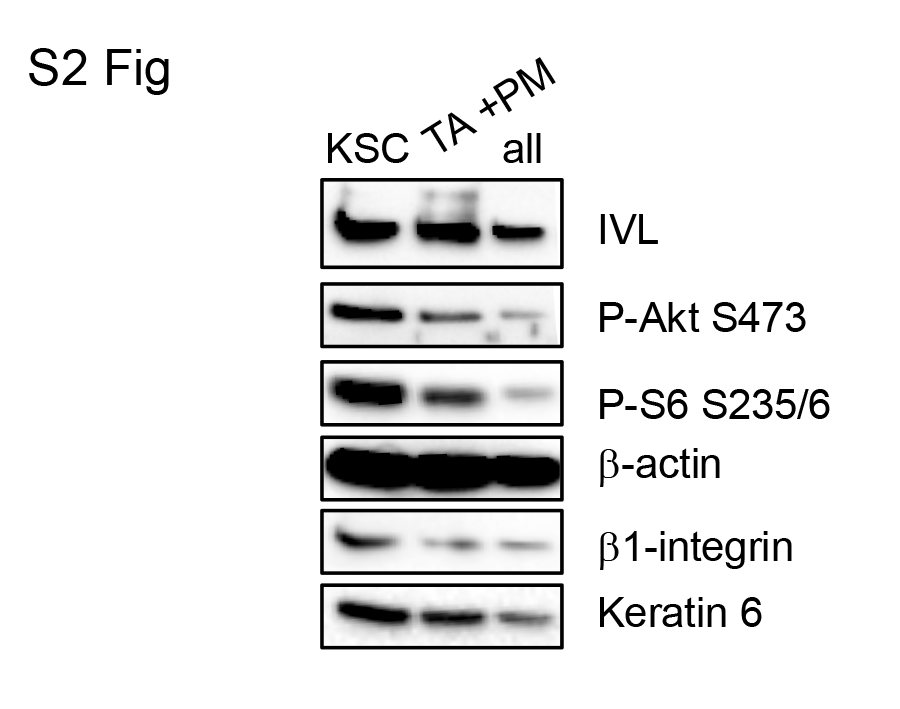

Supplement: S2 Fig — Keratinocytes stem cells (KSC), transient amplifying (TA) and postmitotic (PM) cells were separated according to their ability to adhere to type IV collagen. In addition NHK were seeded in a normal cell culture dish without further separation (all). Protein lysates were subjected to SDS-PAGE and Western blotting with the indicated antibodies. (TIF) [file pone.0180853.s002.tif]

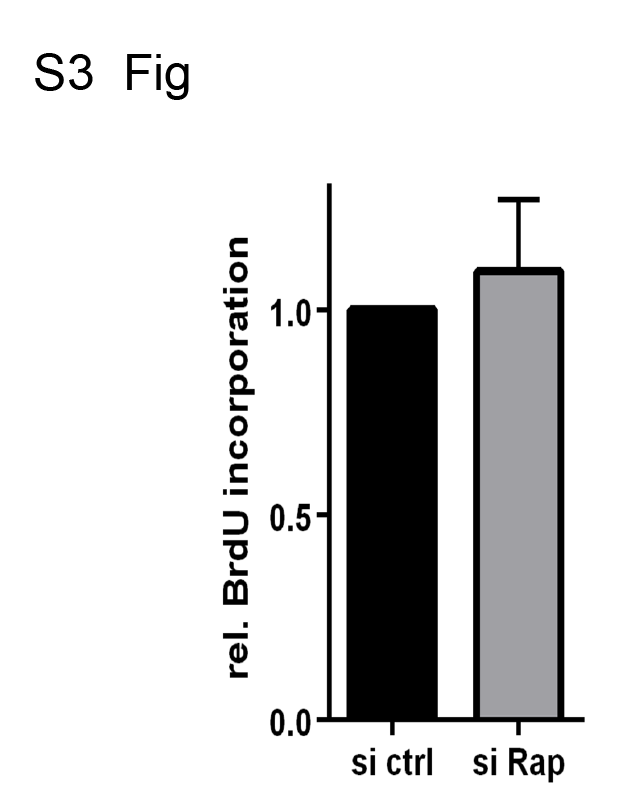

Supplement: S3 Fig — HaCaT cells were reverse-transfected with siRNA targeting Raptor or control siRNA and seeded in 96 well plates. After 72h proliferation was quantified using a BrdU assay. Graph presents mean ± SEM (n = 6). (TIF) [file pone.0180853.s003.tif]

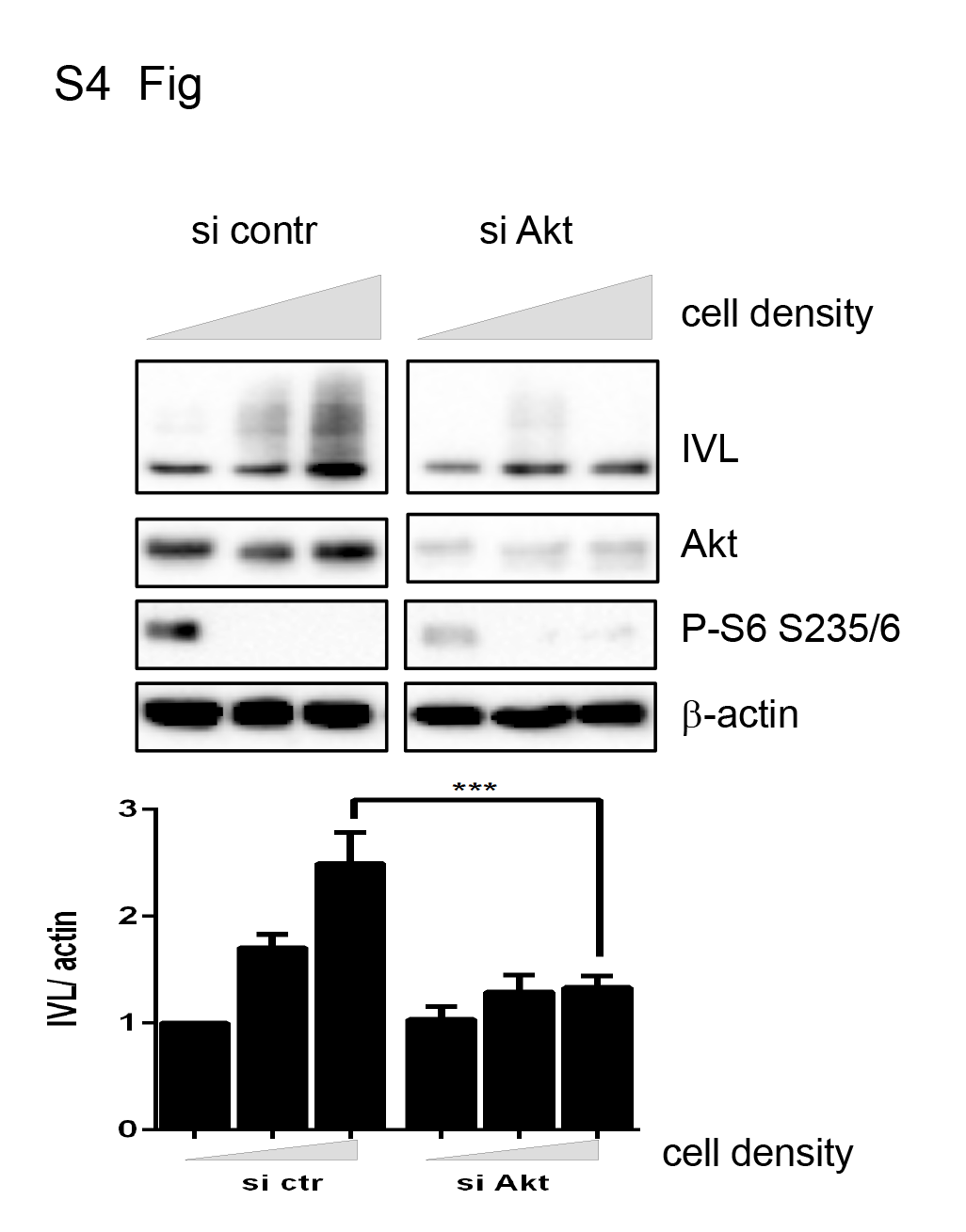

Supplement: S4 Fig — HaCaT cells were reverse-transfected with siRNA specific for Akt or a siRNA control (si contr) and differentiation was induced by post-confluent growth for 72h. Protein lysates were analyzed by Western blotting with the indicated antibodies. Below each blot a quantification of n≥ 3 similar blots is shown. Statistical significant differences between control and knockdown cells of the same density were calculated with one-way ANOVA and Bonferroni multiple comparison (****p ≤0.0001). (TIF) [file pone.0180853.s004.tif]

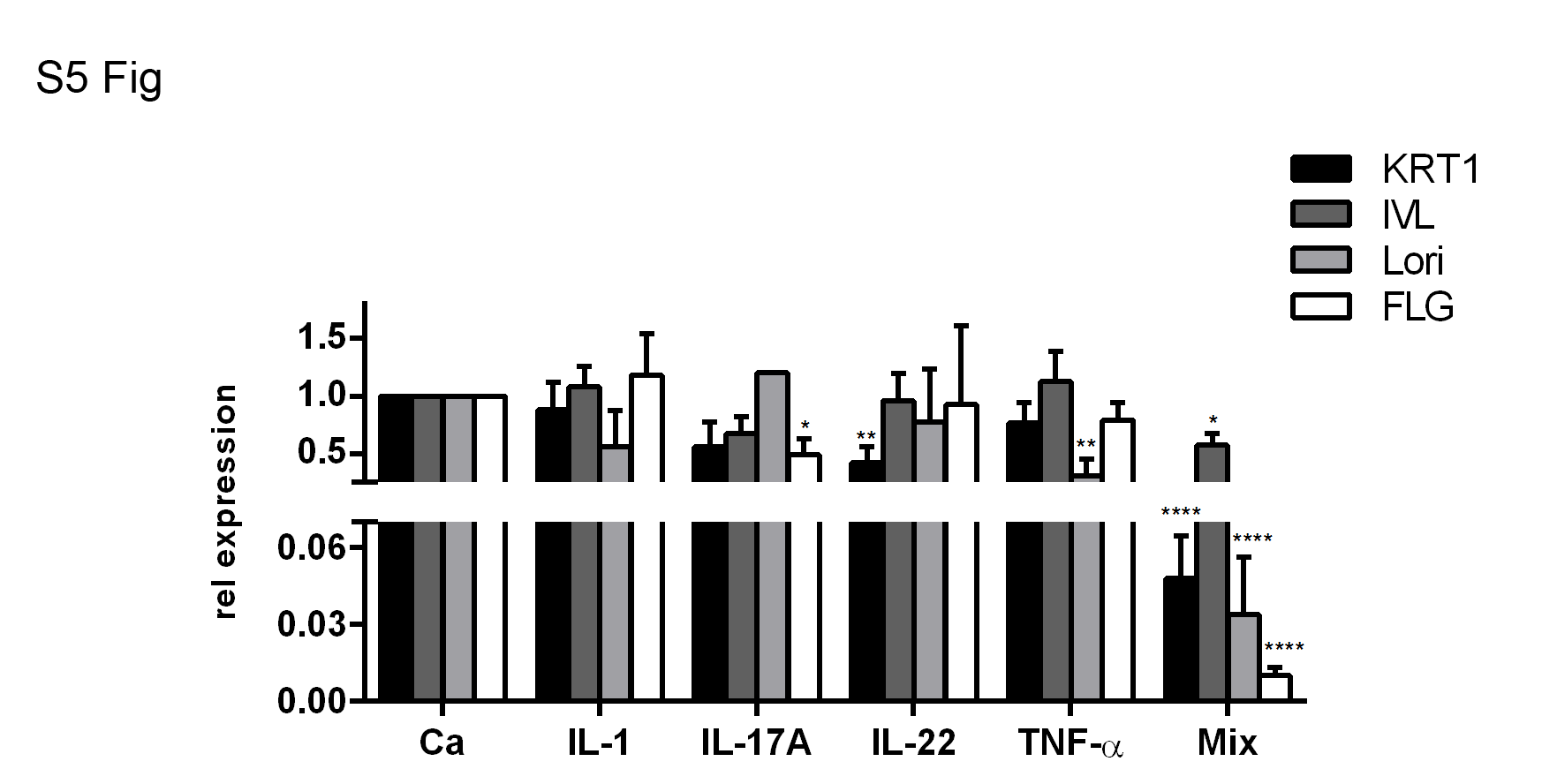

Supplement: S5 Fig — NHK cells were seeded and 24h later, differentiation was induced by the addition of 2 mM CaCl2 in the presence of 20 ng/ml of IL-1β, IL-17A, IL-22 or TNF- α or a mix of IL-1 β, IL-17A and TNF- α. After 72h RNA was isolated and quantitative RT-PCR was performed to measure expression of the indicated differentiation markers. Graph present mean ± SEM (n = 4–8). Statistical significant difference between Ca2+ and the cytokines was calculated with one-way ANOVA and Bonferroni multiple comparison (*p≤ 0.05, **p≤0.01, ****p ≤0.0001). (TIF) [file pone.0180853.s005.tif]

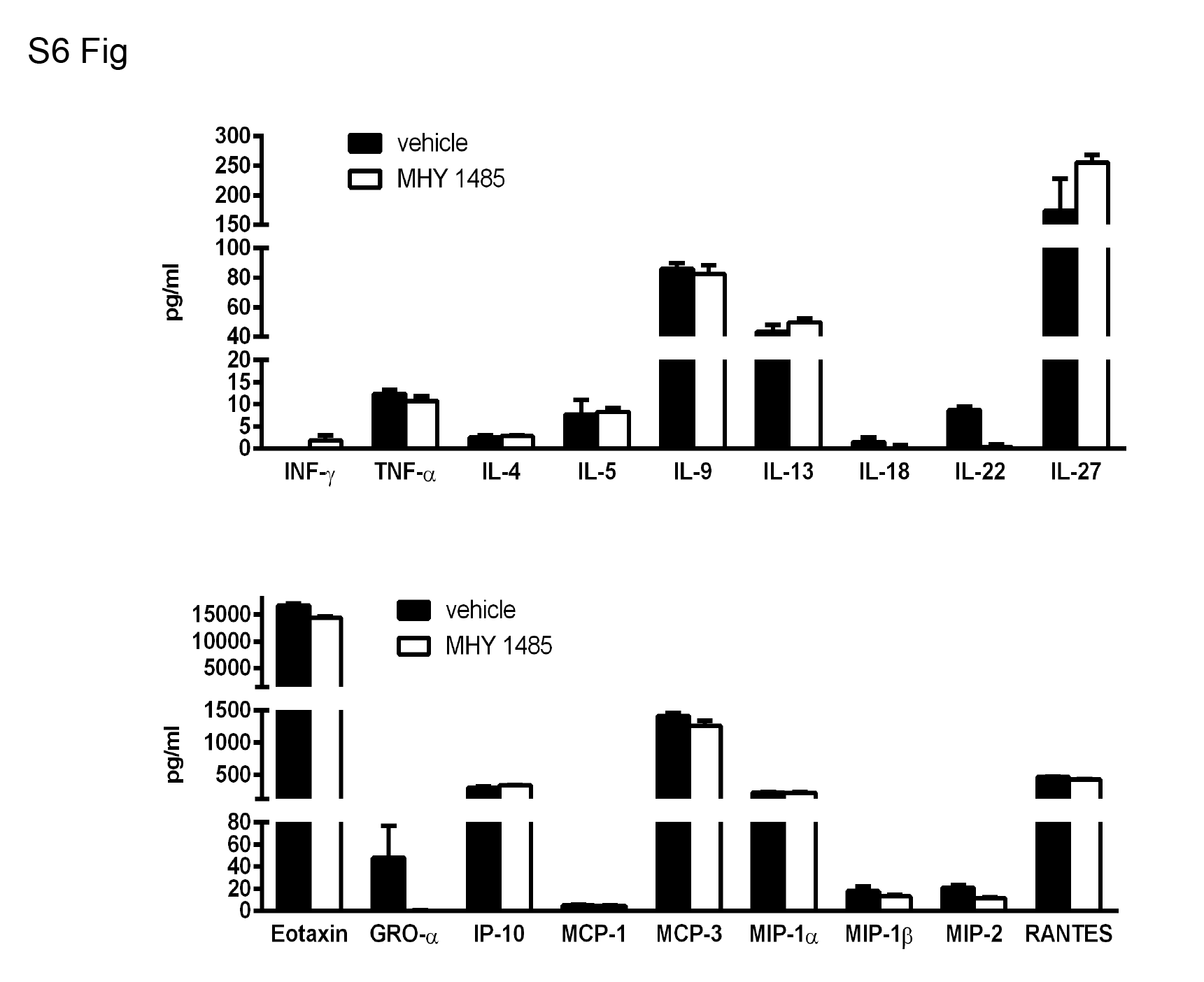

Supplement: S6 Fig — Mice were treated, as described in Fig 6D. At the end of treatment regimen, serum samples were collected and analyzed for protein expression of 26 cytokines and chemokines using multiplex bead immunoassay. IL-17, IL-23, IL-12, IL-1 β, IL-10, IL-6 and GM-CSF levels were not detectable. Data shown are from one experiment, with n = 2–3 mice per treatment group. (TIF) [file pone.0180853.s006.tif]
